# Phantomless calibration of CT scans for hip fracture risk prediction *in silico*: Comparison with phantom-based calibration

**DOI:** 10.1371/journal.pone.0305474

**Published:** 2024-06-14

**Authors:** Julia A. Szyszko, Alessandra Aldieri, Antonino A. La Mattina, Marco Viceconti

**Affiliations:** 1 Medical Technology Lab, IRCCS Istituto Ortopedico Rizzoli, Bologna, Italy; 2 Department of Industrial Engineering, Alma Mater Studiorum—University of Bologna, Bologna, Italy; 3 Polito^BIO^Med Lab, Department of Mechanical and Aerospace Engineering, Politecnico di Torino, Torino, Italy; Ural Federal University named after the first President of Russia B N Yeltsin Institute of Physics and Technology: Ural’skij federal’nyj universitet imeni pervogo Prezidenta Rossii B N El’cina Fiziko-tehnologiceskij institut, RUSSIAN FEDERATION

## Abstract

Finite element models built from quantitative computed tomography images rely on element-wise mapping of material properties starting from Hounsfield Units (HU), which can be converted into mineral densities upon calibration. While calibration is preferably carried out by scanning a phantom with known-density components, conducting phantom-based calibration may not always be possible. In such cases, a phantomless procedure, where the scanned subject’s tissues are used as a phantom, is an interesting alternative. The aim of this study was to compare a phantom-based and a phantomless calibration method on 41 postmenopausal women. The proposed phantomless calibration utilized air, adipose, and muscle tissues, with reference equivalent mineral density values of -797, -95, and 38 mg/cm^3^, extracted from a previously performed phantom-based calibration. A 9-slice volume of interest (VOI) centred between the femoral head and knee rotation centres was chosen. Reference HU values for air, adipose, and muscle tissues were extracted by identifying HU distribution peaks within the VOI, and patient-specific calibration was performed using linear regression. Comparison of FE models calibrated with the two methods showed average relative differences of 1.99% for Young’s modulus1.30% for tensile and 1.34% for compressive principal strains. Excellent correlations (R^2^ > 0.99) were identified for superficial maximum tensile and minimum compressive strains. Maximum normalised root mean square relative error (RMSRE) values settled at 4.02% for Young’s modulus, 2.99% for tensile, and 3.22% for compressive principal strains, respectively. The good agreement found between the two methods supports the adoption of the proposed methodology when phantomless calibration is needed.

## Introduction

Osteoporosis is a systemic metabolic disease characterised by the disruption of bone microarchitecture and bone quality due to an imbalance in the bone remodelling physiological process. Osteoporosis mainly affects older adults, especially postmenopausal women [1–4], and it currently represents one of the most common bone pathologies. Its main clinical manifestation is represented by fragility fractures, most commonly at the hip or vertebrae [5]. Fragility fractures are very severe events, associated with increased morbidity and mortality, and represent a heavy burden for the healthcare system [4, 6, 7]. In addition to chronic pain, osteoporotic fractures also cause loss of mobility and loss of independence, leading to a decreased quality of life [5–7]. According to the International Osteoporosis Foundation, the pathology causes more than 9 million fractures worldwide per year [8], and in 2019, 32 million Europeans aged over 50 years were diagnosed with osteoporosis [2]. Due to the growing global life expectancy, the incidence of hip fractures is expected to increase up to 3.5 times by 2050, compared to 1990 [9, 10].

The dual-energy X-ray absorptiometry (DXA) imaging technique is currently considered the gold standard to diagnose osteoporosis [11, 12]. An average measurement of the bone density, called areal Bone Mineral Density (aBMD), is extracted from the DXA image and used to classify subjects as osteoporotic, osteopenic, or healthy. Nevertheless, the predictive accuracy of aBMD has been demonstrated to be limited [13–15], and there have been several instances where fractures occurred in patients not classified as high-risk by this parameter [16, 17]. In this context, patient-specific finite element (FE) models developed from quantitative computed tomography (QCT) data have demonstrated to represent a valid alternative, able to provide a more accurate fracture prediction [18–20]. In fact, FE models can more accurately account for personalised mechanical determinants of fracture, such as patient-specific bone 3D geometry and density distribution [13, 21–23]. In addition, QCT-based FE models can account for different loads and forces [24–26]. These models rely on a heterogeneous mapping of the material properties based on the Hounsfield Units (HU) of the QCT images, where each element of the FE model is assigned a mineral density value dependent on the image local HU [27]. The element-wise density is later employed to compute the corresponding Young’s modulus values from density-elasticity laws available in the literature [28, 29]. The extraction of densities from the CT HU values requires a calibration to be performed, *i*.*e*., the assessment of a mathematical relation between HU and mineral density. The preferred approach adopted to calibrate CT scans is a phantom-based calibration procedure, which involves either placing a calibration phantom in line with the patient during the scan or scanning a phantom offline using the same scanning parameters employed to image the patient [30, 31]. The phantom is composed of multiple components with different known concentrations of calcium hydroxyapatite (HA) or dipotassium phosphate (K_2_HPO_4_). As a result, a linear relationship can be established between HU and mineral density. However, this approach presents some limitations in the clinical practice. On one hand, the inline scanning is associated with increased costs [32, 33] and may lead to artifacts due to the patient’s position and anatomy, potentially causing issues such as beam hardening or partial volume averaging [32–34]. On the other hand, the offline scanning, which requires scanning the phantom separately, could represent a logistical and financial burden for the hospitals [35]. Moreover, many clinical studies carried out to assess the accuracy of these FE models often make use of opportunistic CT scans, *i*.*e*., CT scans that were originally acquired for other clinical purposes [13, 33]. In these cases, phantom scans are commonly not available.

Aiming to overcome this issue, so-called phantomless calibration approaches have been proposed, which allow the calibration of CT scans without a phantom. Usually, these approaches employ internal patient tissues in place of the components of the phantom. Different tissue combinations can be found in the literature for this purpose, such as air and adipose tissue [36]; muscle and adipose tissues [34, 37]; air, adipose, and muscle tissues [38, 39]; air, aortic blood, and muscle tissues [40]; air, aortic blood, cortical bone, and muscle [33]. Fixed reference density values are hypothesized *a priori* for the selected tissues, and the calibration is performed by extracting the corresponding HU from the scan. In the literature, different approaches identifying the reference values have been presented. Michalski *et al*. [33] proposed a phantomless calibration that used air, adipose tissue, aortic blood, skeletal muscle and cortical bone as references estimating their density values from their mass attenuation coefficients according to [41]. Winsor *et al*. [40], on the other hand, adopted air, muscle and aortic blood as reference tissues, assigning nominal density values provided by the National Institute of Standards and Technology [42] to them. While those studies explored different tissues combinations, including aortic blood, a common limitation of these methods was represented by the manual and therefore user-dependent selection of volumes of interest where the specific tissues were contained. In addition, given that the average HU values for aortic blood fall within the range of values for skeletal muscle [40], the choice of both these reference tissues poses reproducibility and robustness challenges in phantomless calibration. Other studies [36, 38, 39], instead, determined reference density values for the chosen reference tissues by taking advantage of an inline phantom-based calibration. More in detail, in [38, 39] air, adipose tissue and skeletal muscle were employed as references to calibrate femur CT scans of metastatic patients acquired with 120 kVp tube voltage, 220 mA tube current, and 3 mm slice thickness. In [36], instead, phantom-based determined reference values of air and adipose tissues were employed to carry out phantomless calibration used for the prediction of femoral strength. In those works, a phantomless calibration procedure was proposed and density values for the selected reference tissues were also provided. Herein, CT images of post-menopausal women cohort were available along with the corresponding offline phantom-based calibrations. In our efforts to replicate prior phantomless calibration methodologies on our postmenopausal women cohort though, including those outlined by Eggermont *et al*. [38], we came across significant differences in Young’s modulus values derived from the phantom-based calibration available compared to those derived from the phantomless calibration performed adopting the reference density values originally derived there for metastatic patients [36]. Recognizing the need for calibration parameters better suited to our cohort, and analogous phantomless calibration procedure is here proposed, where reference human tissues to be employed in the phantomless calibration are selected and their reference density values identified through phantom-based calibrations across multiple subjects and CT acquisition parameters. The aim of this study was to develop an automatic phantomless calibration methodology to be integrated into an existing FE pipeline for hip fracture risk prediction presented in [18, 43]. In this context, the comparison between the phantom-based and phantomless calibrated FE models is also presented. Our objective was in fact to ensure that our developed method could be robust and easily reproducible by other researchers in the field.

## Materials and methods

### QCT data

For the purpose of this study the QCT scans of 34 postmenopausal female subjects (age 67 ± 8 years, range: 56–84 years; height 160 ± 7 cm, range: 143–180 cm, weight 66 ± 13 kg, range: 39 ± 100 kg) were selected from the Hip-Fracture Validation Collection, freely available at [44]. The collection comprises the CT scans of the femurs of 101 women (age 69 ± 9 years, range: 56–86 years; height 160 ± 7 cm, range: 134–180 cm, weight 65 ± 12 kg, range: 39–100 kg) resident in Emilia Romagna region, collected at Rizzoli Orthopaedic Institute (IOR) from 1999 to 2016 for surgical planning of hip arthroplasty (with approval from Istituto Ortopedico Rizzoli Ethics Committee Area Vasta Emilia Centro CE AVEC 731/2020/Oss/IOR). No consent form for the processing of personal data used for clinical activity for research purposes was applicable, due to article 110 bis paragraph 4 of the Nuovo Codice Privacy D.lgs. 196/2003, updated to D.lgs. 101/2018 regarding the Italian Scientific Institute for Research, Hospitalization and Healthcare (IRCCS). These scans were obtained using GE HiSpeed CT/I (GE Healthcare, Milwaukee, WI) scanner with tube current variations in the 170–200 mA range and slice spacing variations in the 1–2 mm range. The remaining scan parameters were kept constant: tube voltage of 120 kVp, slice thickness of 3 mm, and "bone" reconstruction kernel. In addition, the QCT scans of further 7 female subjects (age 73 ± 6 years, range: 66–82 years; height: 163 ± 4 cm, range: 160–169 cm; weight: 63 ± 13 kg, range: 50–89 kg), collected at Rizzoli Orthopaedic Institute (IOR) in 2023, were also included in the present study. Ethical approval for these subjects was obtained from the Istituto Ortopedico Rizzoli Ethics Committee Area Vasta Emilia Centro CE AVEC 453/2023/Sper/IOR, and all subjects provided written informed consent. These scans were acquired using a GE Discovery CT scanner (GE Healthcare, Milwaukee, WI), with tube current variations in the 100–140 mA range. Other scan parameters remained constant: tube voltage of 120 kVp, slice thickness of 1.25 mm, slice spacing of 1.25 mm, and "bone" reconstruction kernel. Detailed CT scanning settings for all the subjects included in the study are provided in S1 File. The included 41 subjects were selected because scans of a QRM-ESP (QRM GmbH, Germany) calibration phantom were available acquired with matching CT acquisition parameters, as explained in more detail in the following. In order that the phantomless calibration pipeline proposed could be validated, the 41 subjects included were divided into two groups: Group 1, which consisted of 24 subjects representing the largest subgroup with uniform CT parameters, and Group 2, which comprised 17 subjects scanned with tube currents ranging from 100 to 190 mA and with two different CT scanners. Table 1 displays the scan parameter details.

**Table 1:** CT acquisition parameters of the 41 subjects analysed divided into Group 1 and Group 2.

Group	Number of subjects	CT scanner	Tube voltage (kVp)	Tube current modulation (mA)	Slice thickness (mm)
Group 1	24	GE HiSpeed CT/I	120	200	3
Group 2	4	GE HiSpeed CT/I	120	180	3
	2	GE HiSpeed CT/I	120	170	3
	4	GE HiSpeed CT/I	120	190	3
	5	GE Discovery CT	120	100	1.25
	2	GE Discovery CT	120	140	1.25

The original CT scans were anonymized using Synedra View Personal (version 19.0.0.3, Synedra information Technologies GmbH) and subsequently split in order to isolate one of the two femurs, since for this study only one limb for each subject was considered.

### Phantom-based calibration

A QRM-ESP (QRM GmbH, Germany) phantom was employed to carry out the phantom-based calibration. The phantom includes five inserts with different quantities of calcium hydroxyapatite measuring 50, 100, 200, 400, and 800 mg/cm^3^, that cover the range of spongious and cortical bone densities [45]. The phantom was scanned offline using GE HiSpeed CT/I and GE Discovery CT (GE Healthcare, Milwaukee, WI) scanners. A total of 6 CT scans of the phantom were acquired with CT acquisition parameters matching those adopted for the femurs’ CT scans: the tube current was varied in the 100–200 mA range and slice spacing adjusted between 1.25–4.5mm, with slice thickness of either 1.25mm or 3mm, while maintaining a constant “bone” kernel and tube voltage of 120 kVp. Detailed information about the ESP phantom scans is provided in Table 2. For each scan, the phantom inserts were manually segmented, and the corresponding average HU values were computed. Hence, linear regressions between the average HU values and the known density of the phantom inserts were performed to identify the coefficients of the calibration line for each scan. S1 File provides the phantom-based calibration information for each subject included in this study.

**Table 2:** Detailed information of ESP phantom scans with different configurations.

CT settings	Spacing between slices (mm)	Slice Thickness (mm)	Tube Voltage (kVp)	Xray Tube Current (mA)	Kernel	Manufacturer	Manufacturer Model
1	4.5	3	120	180	Bone	GE MEDICAL SYSTEMS	HiSpeed CT/i
2	4.5	3	120	200	Bone	GE MEDICAL SYSTEMS	HiSpeed CT/i
3	4.5	3	120	170	Bone	GE MEDICAL SYSTEMS	HiSpeed CT/i
4	4.5	3	120	190	Bone	GE MEDICAL SYSTEMS	HiSpeed CT/i
5	1.25	1.25	120	100	Bone	GE MEDICAL SYSTEMS	Discovery CT
6	1.25	1.25	120	140	Bone	GE MEDICAL SYSTEMS	Discovery CT

### Phantomless calibration

**Fig 1:**
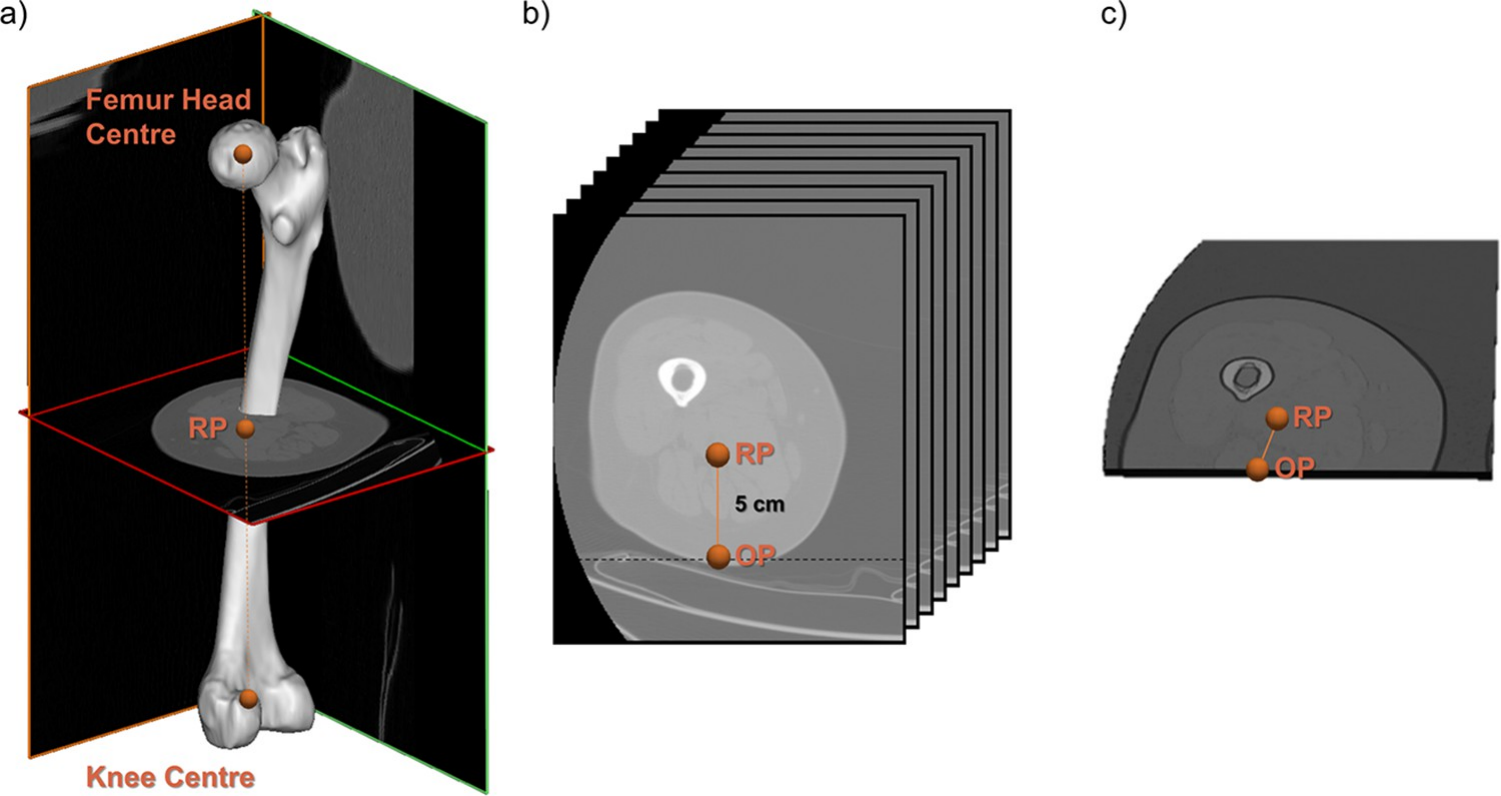
Definition of VOIcut from CT scan slices. (a) RP definition based on the Femur Head Centre and Knee Centre. (b) VOI definition based on RP and OP by removing the portion extending 5cm posteriorly from the RP. (c) VOIcut selection. The visualization of the CT scan was obtained from the data collected during the study, which is available at [42].

The phantomless calibration procedure was based on the methodology reported in [38], where air, adipose, and muscle tissues were employed to carry out calibration. The main steps of the phantomless calibration procedure will be described in detail in the following. First, a reference point (RP) was identified as the average between the femur head and the knee centres (Fig 1A). The CT slice closest to the RP was then selected, and a 9-slice-wide volume of interest (VOI) was defined from that slice in the caudal direction. The selected VOI included air, adipose, and muscle tissues (Fig 1B). In order to eliminate any artifacts, the VOI was restricted by removing the portion extending in the posterior direction, starting from a point (OP) located 5 cm posteriorly to RP (Fig 1C). Since only one limb CT scan was used, no restriction in the medial direction was applied. The volume of interest resulting from this process will be referred to as VOIcut in the following.

**Fig 2:**
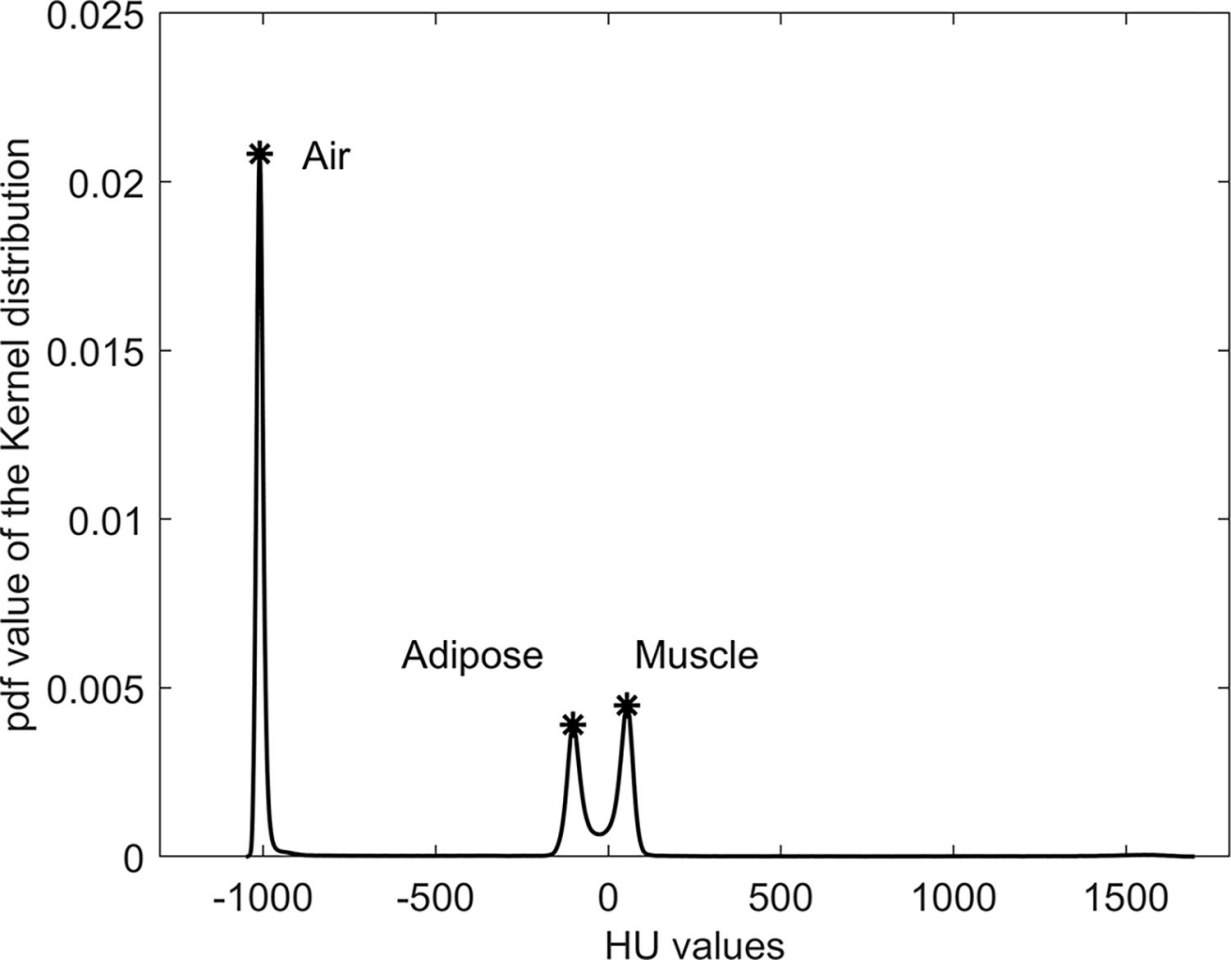
Example of fitted distribution of the HU in the VOIcut, with the extracted peaks of air, adipose and muscle tissues. The "pdf value" on the y-axis represents the probability density function value.

The voxels HU contained in the VOIcut were used to build a histogram, which was later fitted with a kernel distribution using a normal smoothing function based on a 5 HU bandwidth (Matlab, release R2022b, The Mathworks Inc) (Fig 2). The peaks corresponding to air, adipose tissue, and muscle HU reference values could thus be identified. Further details about the kernel distribution fitting procedure are provided in the S1 File.

**Fig 3:**
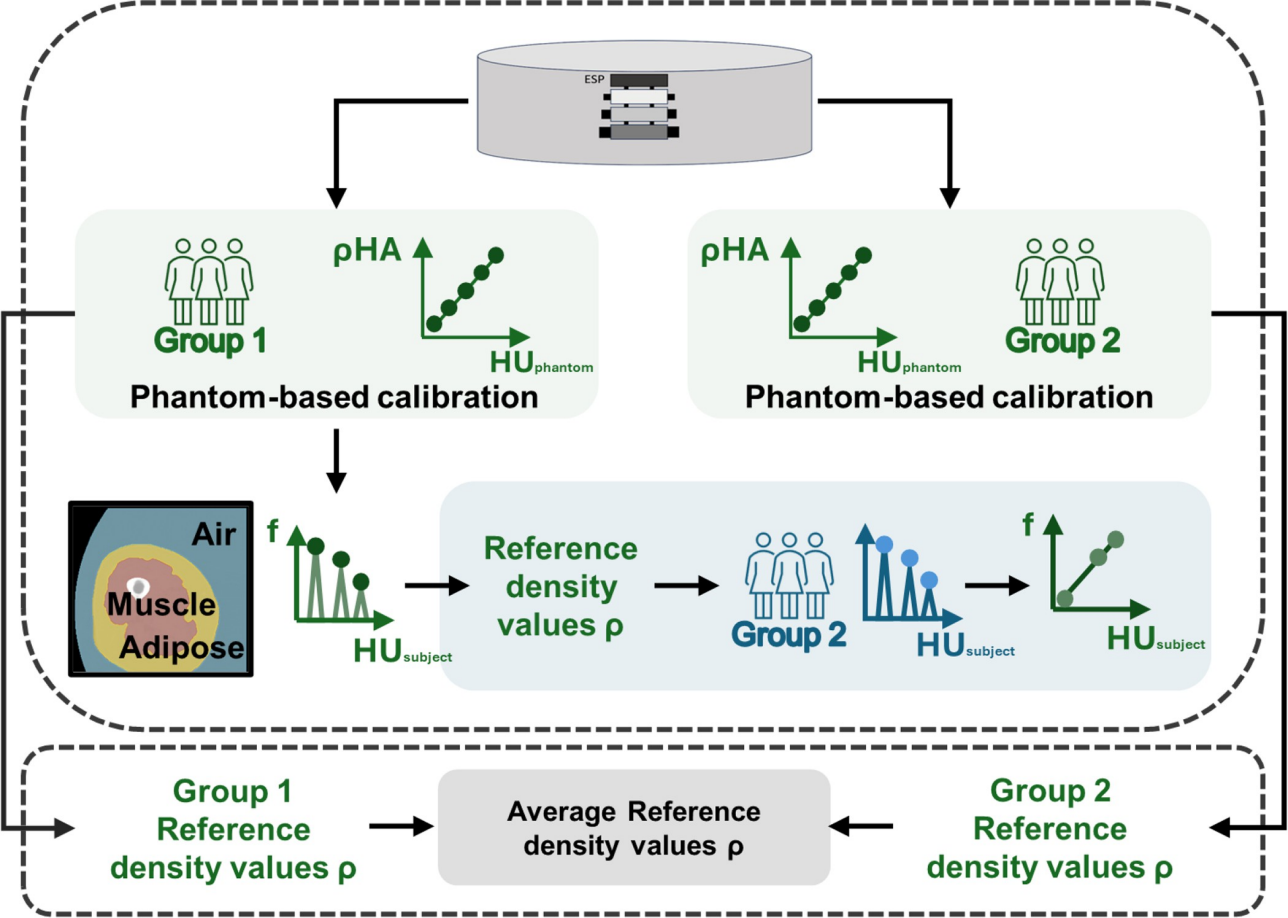
Graphical overview of the study. Upper panel: the calibration phantom was employed to calibrate the CT scans for Group 1 subjects so that reference density values for air, adipose, and muscle tissues could be computed. These reference density values were later employed to calibrate through a phantomless calibration procedure the CT images of Group 2 subjects. Phantom scans were also available to calibrate Group 2 subjects, so that the outcomes of the phantomless and phantom-based calibration methodologies could be compared. Lower panel: phantom-based calibrations of Group 2 subjects were also employed to compute reference values for air, adipose, and muscle tissues to assess potential effects of different CT acquisition parameters on the reference density values. In the figure, ρ_HA_ stands for the density of the phantom, f stands for the distribution of the HU in the VOIcut (pdf value), HU_phantom_ and HU_subject_ stand for HU values extracted from the CT scan of the phantom and subject, respectively.

For each of the 24 subjects in Group 1 the equivalent mineral density values for air, adipose, and muscle tissues was computed by means of the available phantom-based calibration line. Subsequently, their equivalent mineral density values were averaged, resulting in -797, -95, and 38 mg/cm^3^ for air, adipose, and muscle, respectively. These reference values were then adopted in the phantomless calibration process performed on Group 2 subjects, where calibration was performed employing the above-mentioned reference density values previously obtained from Group 1 instead of employing the phantom scans. Aiming to assess whether different CT acquisition parameters could affect the reference density values identified for air, adipose and muscle tissues and obtained for Group 1 employing the phantom-based calibration, those reference density values were also extracted through phantom-based calibration on Group 2, in order that a comparison with the values obtained for Group 1 could be carried out. The overall overview of the procedure implemented is shown in Fig 3.

### FE models

FE models of the femur were built for all 41 subjects included in this study according to the methodology presented in [43] and here briefly summarized. First, the CT scans were segmented using a semi-automatic methodology [46], and the femur geometry was extracted. Subsequently, a 2-mm-sized 10-nodes tetrahedral mesh was built using Ansys ICEM CFD (release 2019R3, Ansys Inc.). Bonemat (V3.2, Istituto Ortopedico Rizzoli, Bologna, Italy) was used to assign heterogeneous linear elastic material properties to the models, and specifically to map the HU-based Young’s modulus values to the mesh elements [28, 29]. For each subject tested, the material properties were mapped using both the phantom-based and the phantomless calibrations on the same mesh. Boundary conditions replicating a sideways fall loading scenario were applied to the models. Specifically, a 1000 N load was applied at the femur head center, and a rigid frictionless contact plane perpendicular to the load direction was created at the greater trochanter. A revolute joint was placed at the knee rotation centre (calculated as the mean location between most medial and most lateral epicondyle points), with the rotation axis along the antero-posterior direction, perpendicular to the load axis. A total of 28 different FE simulations were performed by varying the femur impact pose from 0° to +30° in the medio-lateral plane and -30° to +30° in the antero-posterior plane [26, 43]. Simulations were solved with Ansys Mechanical APDL (Ansys Inc., PA, USA). The principal strain values represented the biomechanical variables of interest. In addition, for each impact pose, the load to failure, i.e. the intensity of the force required to fracture the femur was computed based on principal strains. The region of interest (ROI) chosen for determining load to failure included surface nodes located at the proximal femur (representing 25% of its biomechanical length in the longitudinal direction) [41]. By comparing the so obtained loads to failure with forces acting on the femur upon falling computed with a stochastic analytical model [18, 25, 43] the absolute risk of hip fracture at time 0 (ARF0), where time 0 refers to the time of the QCT scan, was derived [41].

### Comparison metrics

Young’s modulus values of each element of the mesh together with the superficial tensile (*ε*_1_) and the compressive (*ε*_3_) principal strains obtained on the ROI resulting from the two calibration procedures were compared, and the root mean square relative error (RMSRE) computed using the average values of phantom-based and phantomless calibrations as normalizing values:

$$RMSRE=\sqrt{\frac{1}{n}\sum_{i=1}^{n}{\left(\frac{x_{i}-{\hat{x}}_{i}}{\bar{x_{i}}}\right)}^{2}},$$ (1)

where:

■ n is the number of data points.
■ *x*_*i*_ represents the value obtained from phantom-based calibration.
■ ${\hat{x}}_{i}$ represents the value obtained from phantomless calibration.
■ $\bar{x_{i}}$represents the average between phantom-based and phantomless calibration.

Point-to-point comparisons between the phantom-based and phantomless calibrated FE models were also performed for each subject considering principal strains and Young’s moduli. In addition, the differences in Minimum Side-Fall Strength (MSF), i.e., the lowest load to failure across the 28 femoral impact poses, and ARF0 resulting from the phantom-based and phantomless calibrated femur FE models were calculated for each subject.

## Results

Table 3 presents the mean and standard deviation of the reference density values obtained from the phantom-based calibration for air, adipose, and muscle tissue, for both the Group 1 and for all 41 subjects averaged together. Small variations can be observed between the obtained reference density values.

**Table 3:** Reference equivalent mineral density values (in mg/cm^3^) of air, adipose and muscle tissues obtained for the Group 1 and for all 41 subjects.

	Air		Adipose		Muscle	
	Mean (mg/cm^3^)	SD (mg/cm^3^)	Mean (mg/cm^3^)	SD (mg/cm^3^)	Mean (mg/cm^3^)	SD (mg/cm^3^)
Group 1 (24 subjects)	-797	2.6	-95	2.3	38	3.1
All subjects (41 subjects)	-801	4.9	-96	3.1	37	3.1

**Fig 4:**
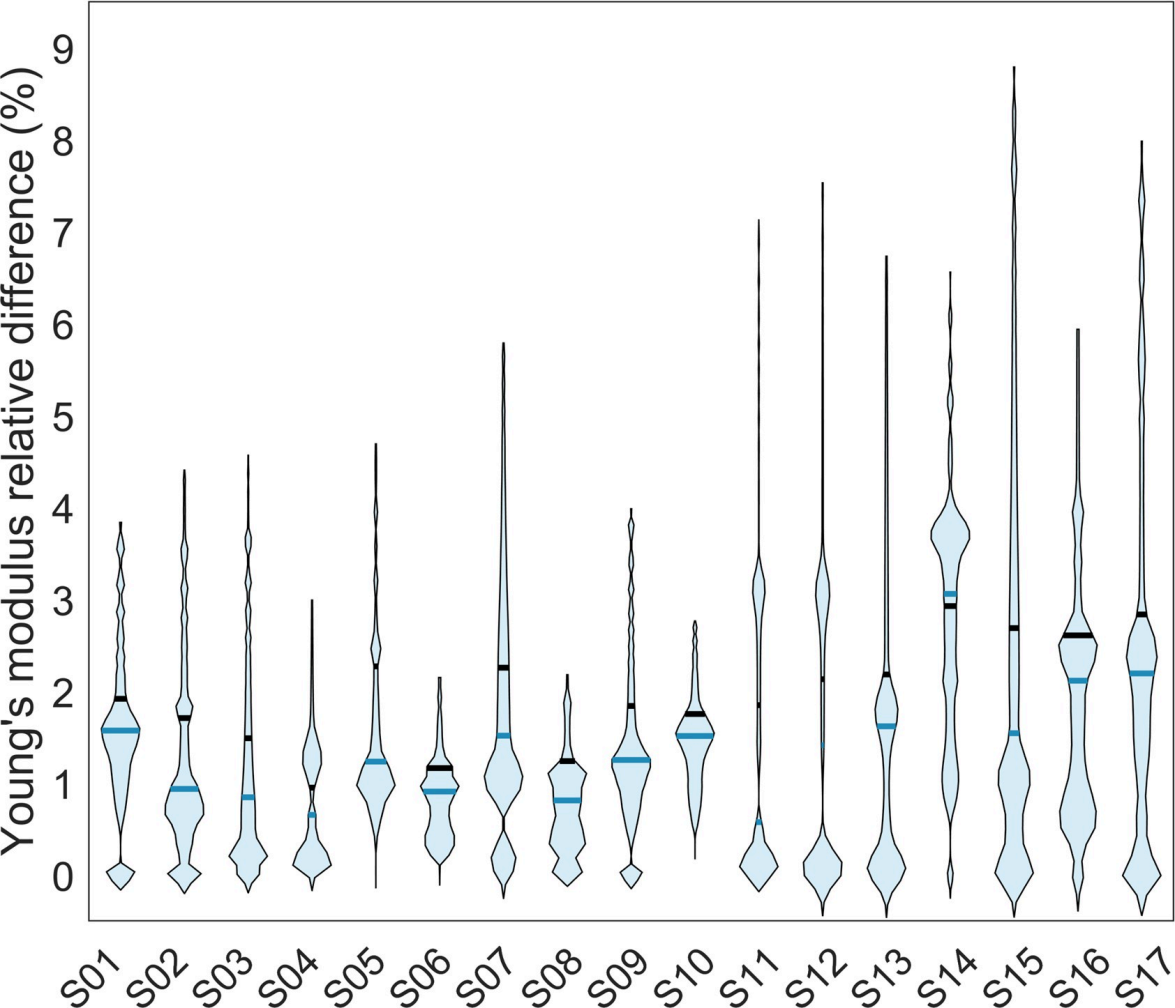
Distribution of element-wise relative differences in Young’s modulus between both calibration methods. Violin plots showing the distributions of element-wise relative differences in Young’s modulus values between phantom-based and phantomless calibration for Group 2 subjects. The solid black line represents the mean value, while the blue solid line represents the median. Outliers, identified using the inter-quartile range method, have been excluded.

The RMSRE computed on Young’s modulus values for Group 2 subjects settled to an average value of 2.86 ± 0.76%, with a maximum of 4.02%. The mean RMSRE computed on superficial principal strains for the same subjects turned out to be, on average, equal to 1.55 ± 0.74%, with a maximum of 2.99% for tensile and 1.61 ± 0.80%, with the maximum being 3.22% for compressive principal strains. Fig 4 shows the violin plots relative to the element-by-element relative differences in the Young’s modulus resulting from the two calibration methodologies for Group 2. There, outliers were excluded using the Interquartile Range (IQR) method, which calculates the range between the first quartile (Q1) and the third quartile (Q3) of the data distribution and removes data points falling beyond a threshold defined as 1.5 times the IQR. The outliers were identified as high errors associated with low Young’s modulus values, primarily attributed to the spongy bone region of the femur and by the femur head typically associated with the low cortical area. Violin plots containing outliers can be found in the S1 File. Element-wise relative differences in Young’s modulus averaged on each subject belonging to Group 2 ranged from 0.95% to 2.93%, with an average value of 1.99%.

**Fig 5:**
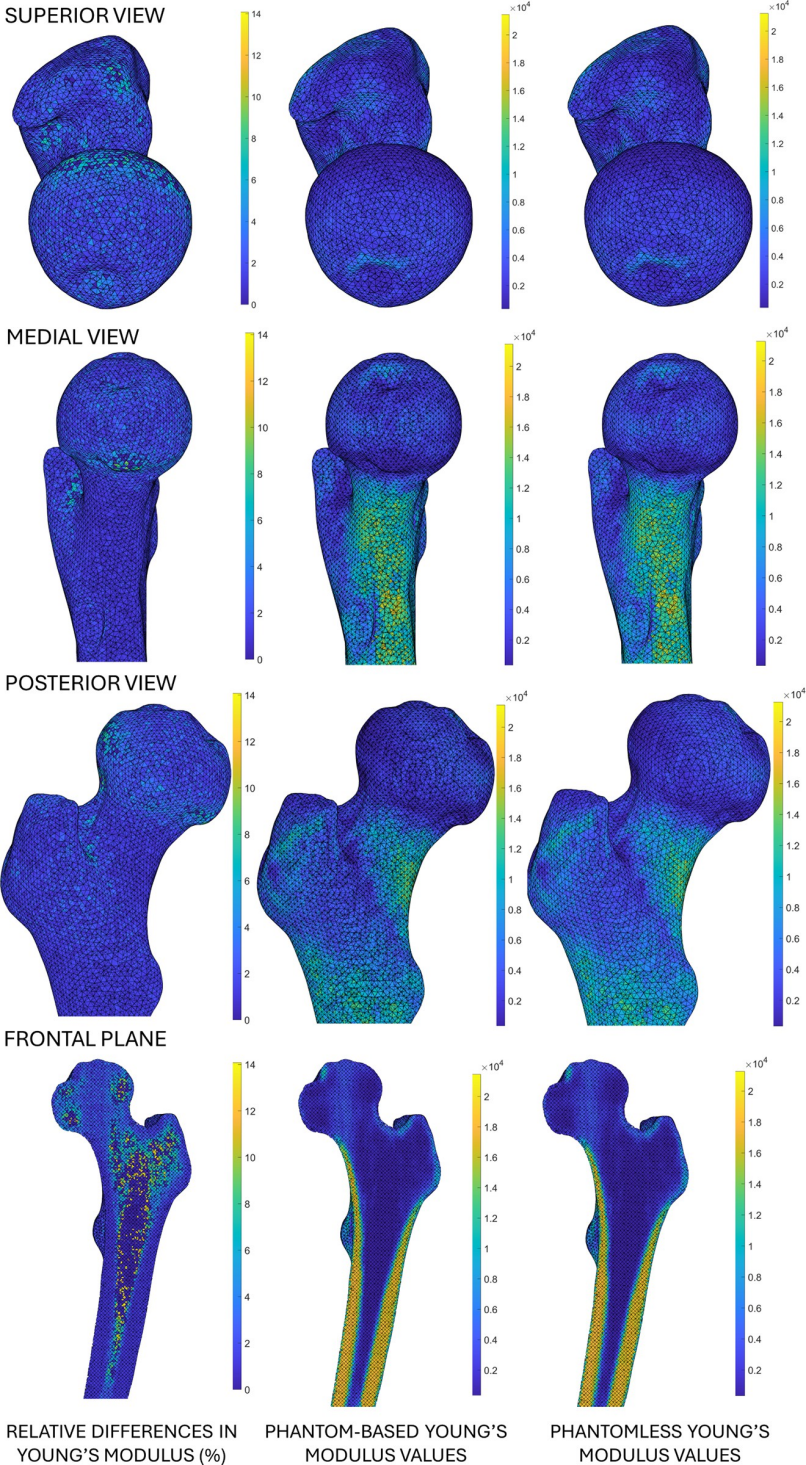
Spatial distribution of relative differences between Young’s modulus values coming from phantom-based and phantomless calibrations. Frontal plane is the mean frontal section from the anterior view of the femur.

Fig 5 shows the spatial distribution of the relative differences in Young’s modulus along with the Young’s modulus values obtained for both methods for one of the analysed subjects (S07). Spatial distributions of relative differences in Young’s modulus are shown for additional subjects in the S1 File.

**Fig 6:**
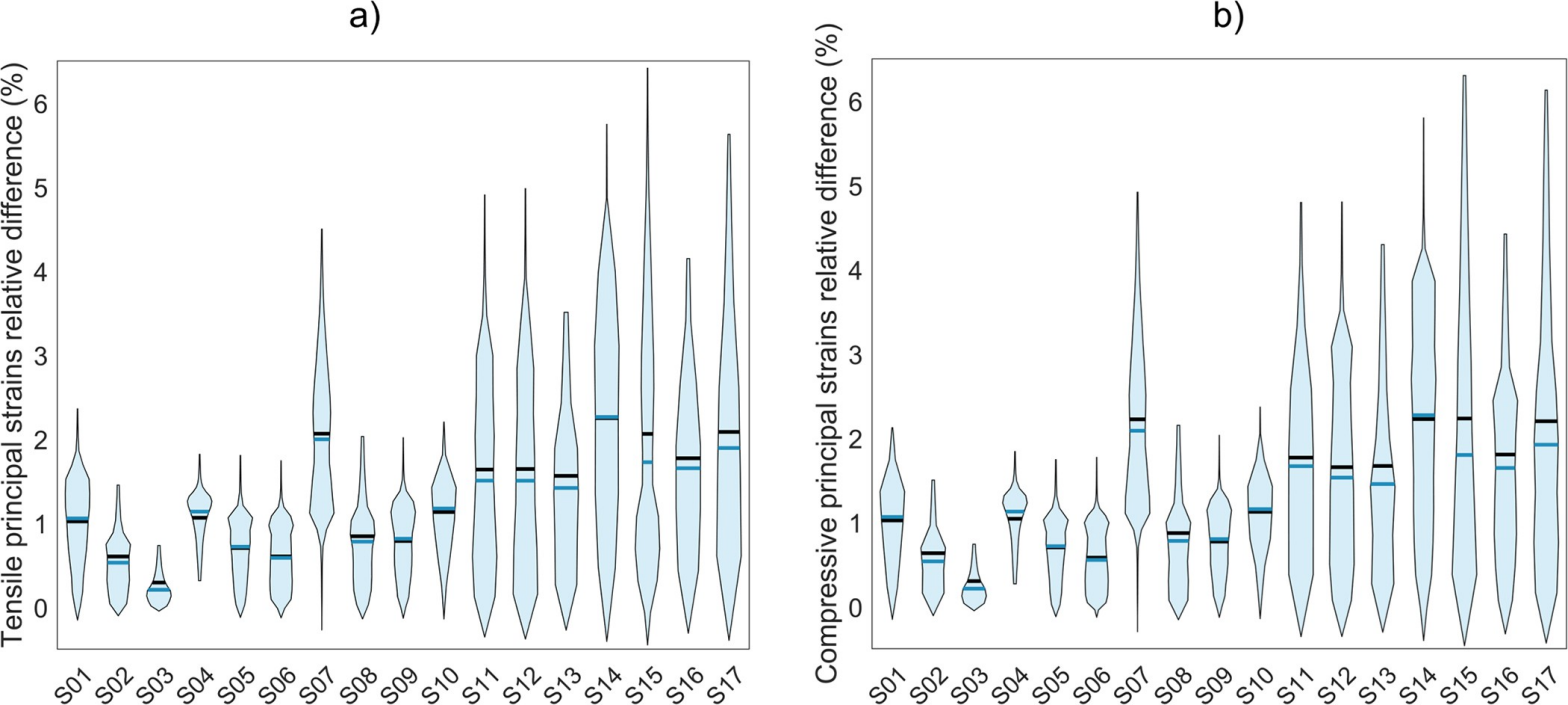
Distribution of point-to-point relative differences in superficial principal strains coming from both calibration methods. Violin plots showing the distributions of the point-to-point relative differences computed on superficial tensile (a) and compressive (b) principal strain values between phantom-based and phantomless calibrations, considering all 28 simulated femur impact poses for each the 17 subjects in Group 2. The solid black line represents the mean, the blue solid line represents the median. Outliers, identified using the inter-quartile range, have been excluded.

The point-to-point relative differences in superficial principal strains averaged on each subject belonging to Group 2 ranged from 0.29% to 2.53%, with an average value of 1.30% for tensile principal strains and from 0.29% to 2.69%, with an average value of 1.34% for compressive principal strains. A more detailed comparison carried out on tensile and compressive principal strains, including all the simulated femur impact poses, can be found in the S1 File. Fig 6 shows the distribution of the point-to-point relative differences between the phantom-based and phantomless calibration for the superficial tensile (Fig 6A) and compressive (Fig 6B) principal strains, respectively. Outliers identified using the inter-quartile range method have been excluded. Violin plots containing outliers can be found in the S1 File.

**Fig 7:**
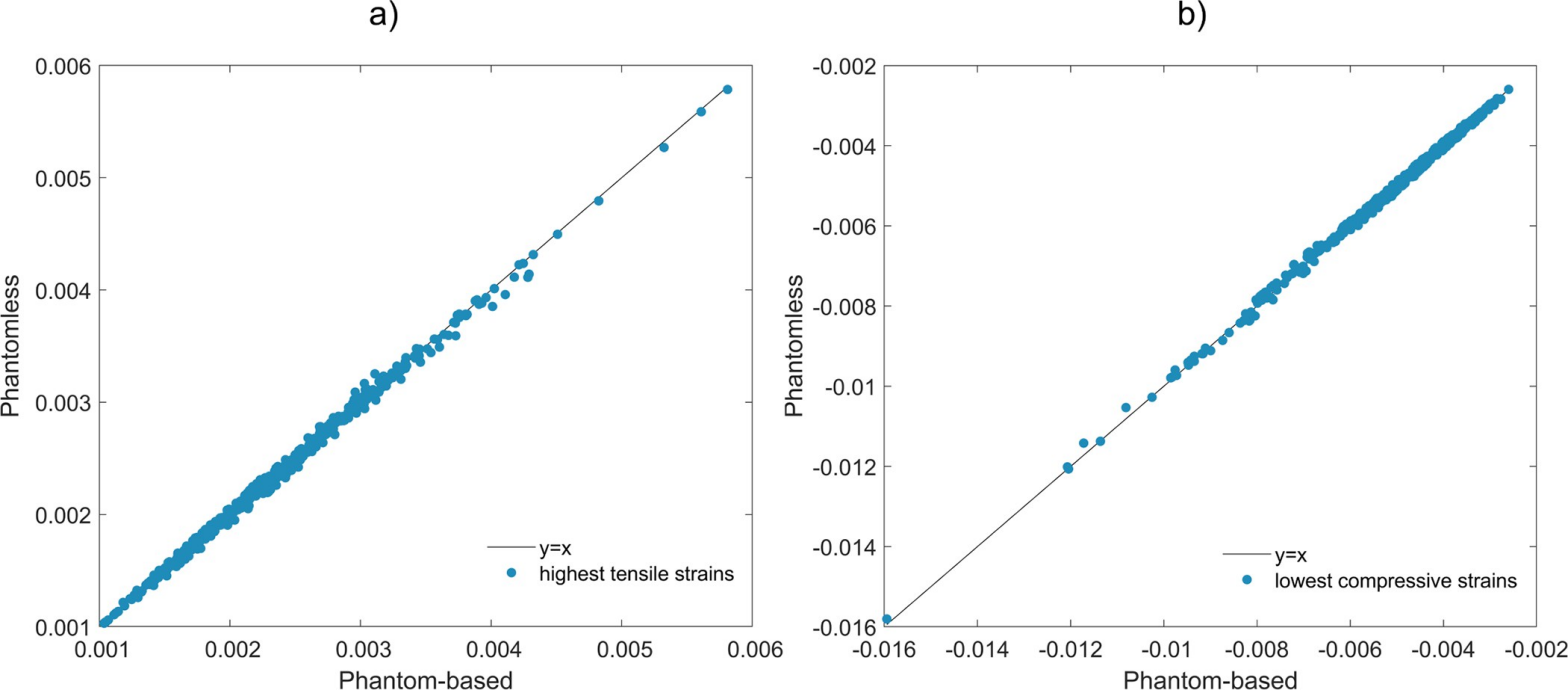
Linear regressions plots between phantom-based and phantomless calibrations-derived highest tensile and lowest compressive strains. The highest superficial tensile (a) and the lowest superficial compressive (b) principal strains for each of the 28 simulated impact poses for each of 17 subjects contained in Group 2 (R^2^ > 0.99).

The correlation between phantom-based and phantomless calibration-derived Young’s modulus and principal strains was excellent (R^2^ > 0.99). In Fig 7 the scatter plot comparing phantom-based and phantomless calibrations-derived highest superficial tensile and lowest superficial compressive strains is depicted. Specifically, the highest superficial tensile (a) and the lowest superficial compressive (b) principal strains for each simulated impact pose (28 angles) and Group 2 subjects are shown.

The relative differences computed for MSF ranged from 0.08% to 2.41%, with an average value of 0.98%. S1 File reports the MSF values along with the associated failure load angles for the phantom-based and phantomless calibrated FE models. Similarly, the absolute differences computed for ARF0, which can be found in S1 File for Group 2, ranged from 0.01% to 1.87% with an average of 0.59%.

## Discussion

When building a patient-specific QCT-based FE model of the femur, heterogenous material properties are assigned to the mesh elements based on the local density information contained in the CT scan. In fact, a number of mathematical laws have been proposed and validated in the literature, which relate density and Young’s modulus values [28, 29, 47]. Nonetheless, in order for any of those relationships to be used, density needs to be estimated from the HU values. Calibration, establishing the HU-density relation, is in fact a crucial step in QCT-based FE modeling. Typically, calibration involves scanning a phantom with known density components, so that a mathematical relationship can be established between the average HU and the known density of the components themselves. However, the availability of a phantom to be scanned as well as the availability of phantom scanned with CT acquisition parameters matching exactly the CT scan of a subject are not straightforward and may be challenging. Therefore, a phantomless calibration procedure, *i*.*e*., an approach able to relate HU and density without the need for a phantom, but uniquely based on the subject’s CT scan, would be pivotal to extending the usability of these patient-specific models. In this context, the primary objective of this study was to develop and evaluate an automatic phantomless CT calibration approach according to the methodology proposed in [38]. The developed procedure only requires input data in the form of a CT scan and predefined anatomical landmarks such as head femur center and knee center. The output of the procedure is the calibration line, which is generated automatically. With this aim, in this work the outcomes of subject-specific QCT-based FE models built starting from phantom-based and phantomless calibration procedures have been compared to assess the reliability of the phantomless calibration approach.

Our study involved a cohort of 41 postmenopausal women whose CT scans were obtained with different scanners, tube current values (100–200 mA), slice thickness (1.25–3 mm) and spacing between slicing (1–2 mm). Density values for the air, adipose and muscle tissues selected as references to be adopted for future calibrations were defined based on the phantom-based procedure for 24 subjects whose scans had been acquired with the same CT parameters, and which formed so called Group 1. The phantomless calibration method was then validated on 17 subjects referred to as Group 2, for whom phantom-based calibration was also available. Nevertheless, aiming to assess whether the identified reference density values were robust enough, those were computed through phantom-based calibration also considering Group 2 subjects. Although some variations could be observed between air, adipose and muscle reference density values extracted considering Group 1 only or Group 1 and 2 together, these differences were minimal and did not significantly impact the calibration process and the reliability of the obtained results. The density values obtained for Group 1 were -797, -95, and 38 mg/cm^3^ for air, adipose, and muscle tissues, respectively. When including all subjects, these values shifted slightly to -801, -96, and 37 mg/cm^3^. In the studies presented by Eggermont *et al*. [38] and Ataei *et al*. [39], different reference values for the same tissues were reported: -840, -90, 30 and -838, -86, and 35 mg/cm^3^ for air, adipose, and muscle tissues, respectively. These differences in the reference density values may be attributed to several factors. Firstly, our study exclusively focused on postmenopausal women, whereas their cohorts included both elderly men and women, including premenopausal women, diagnosed with bone metastases. As literature suggest, the density of adipose, and muscle tissues may vary, depending on gender, age or health conditions [48–50], and therefore adjustments to the here proposed reference density values may be necessary for accurate results. Moreover, while in our study reference density values identification was based on an offline phantom-based calibration, in [38, 39] an inline calibration method was adopted by scanning the patient atop of the calibration phantom. Previous studies have shown that scanning the patient atop of the phantom might cause artifacts due to the patient’s position and anatomy, thus resulting in issues such as beam hardening and scatter, partial volume averaging, or a decrease in HU values in the phantom due to the gap between the patient and the phantom [33, 34, 36, 37]. Offline calibration, on the other hand, eliminates these variables, providing a calibration process free from the influence of patient-specific factors during the phantom scanning procedure. On the other hand, the scanning of the phantom separately from the patient may not fully account for beam hardening effects related to patient size and anatomy [51]. It is known that higher amounts of soft tissue tend to absorb lower energy X-ray photons, leading to beam hardening and variability in HU measurements [33].

Considering the Young’s modulus differences here obtained by comparing the phantom-based and the phantomless calibrated FE models for Group 2, limited point-to-point relative differences were observed. Notably, the most significant local point-to-point differences were associated with low-density values located in the spongy bone tissue (Fig 5). These variations did not substantially impact the FE predicted femur strength, which was determined based on superficial principal strains. Furthermore, the maximum RMSRE calculated on Young’s modulus was less than 5%, indicating small variations between both calibration methods. In a comparable study [39], the relative differences between the Young’s modulus obtained from the mean bone density values for the cortical and trabecular bone for two calibration methods resulted in 5.47% and 4.6%, respectively. The point-to-point relative differences in superficial tensile and compressive principal strains exhibited low errors, with the highest mean differences of 2.53% and 2.69%, respectively. Importantly, in our study, the correlation between the principal strains resulting from the two calibration methods was notably high, with R^2^ > 0.99, thus affirming a strong agreement in the strains prediction. Additionally, the mean relative differences in the MSF resulted in 0.97% with the highest being 2.41%. These findings agree with similar studies [38, 39], where relative differences between the mean failure load turned out to be 0.16% and 6.96%, respectively. There, a high correlation emerged between failure loads of phantom-based and phantomless calibrated FE models, with R^2^ = 0.94 and R^2^ = 0.83 for [38, 39], respectively. Similarly, our study showed an excellent correlation between failure loads, with R^2^ > 0.99. Winsor *et al*. [40] reported absolute differences of 6.9% and 3.9% for femur strength and femoral bone density, respectively, while comparing the inline phantom-based and phantomless method. Nevertheless, the differences computed on femur strength and femoral bone density increased significantly when comparing FE models calibrated with offline phantom-based and phantomless calibration methodologies (22% and 17% relative differences for femur strength and femoral bone density, respectively). In our simulation outcomes the absolute differences in ARF0 for all subjects remain below 2%. Crucially, these differences would not significantly impact the classification of the subjects as at high or low fracture risk, as the ARF0 threshold for classification previously obtained for the full cohort was set at 39.98%. As a whole, despite the different FE pipelines and reference tissue combinations adopted, the outcomes obtained in our study closely agreed with those reported in studies where CT phantomless calibration was also carried out.

There are some limitations to this study, which are reported in the following. First of all, the assumption of consistent muscle and adipose tissues density among all subjects was made, which is a strong hypothesis required when a phantomless calibration procedure is developed. While we acknowledge the potential impact of sarcopenia on muscle density for instance, we tentatively assumed similar muscle states across our study participants, given the narrow age range of postmenopausal women included in our sample. However, we recognize that different pathologies may introduce variability in muscle density, and future studies might provide valuable insights into this aspect of our research. Notably, despite these potential discrepancies, the average relative differences observed on Young’s modulus and principal strains for all subjects in Group 2 remained below 4%. This suggests that while individual variations in effective density might be present, they may not substantially impact the overall outcomes. Moreover, while our cohort is small and focused, it was intentionally selected to address research dedicated to fracture risk prediction in postmenopausal osteoporotic subjects. Nonetheless, adjustments to the proposed reference density values may be necessary for accurate results in different populations. From a general perspective, our study also provides the framework to recalculate such reference density values for different populations if needed.

In addition, when applying the here presented methodology to very skinny subjects with minimal adipose tissue, issues might arise in detecting the adipose tissue peak due to its reduced presence in CT images. Although our methodology employs a histogram analysis with a narrow bin width (1 HU) to identify subtle tissue peaks, accuracy may be compromised in instances of extremely low BMI. On the other hand, one of the subjects considered in this study actually had a BMI value of 14.31, which is classified as underweight. In that case tissue peaks could be successfully identified. However, further investigations about the impact of subject anatomy and composition on the calibration process would be of interest, especially across different populations. Moreover, although the subjects analysed here were scanned with two different CT scanners and with varying CT acquisition parameters (e.g. tube current, slice spacing, and slice thickness), it is important to point out that tube voltage never varied. Previous studies have shown that alterations in tube voltage have the most significant impact on HU [39, 52]. Slightly higher errors were observed for subjects S11-S17 compared to other subjects in Group 2. While these errors indicate minimal impact on our overall results, further studies including more subjects and CT scanners would be beneficial to assess inter-scanner and cohort variability and its impact on the phantomless calibration. Therefore, a more comprehensive analysis involving diverse tube voltage settings and additional CT scanners would be essential to enhancing the robustness and consistency of the here achieved results. Another aspect to be considered is also the time elapsed between the phantom and the subjects scans, which in our case varied from a few months to up to a few years: this variability in timing may have impacted the stability of HU values among scans, and potentially have affected calibration accuracy.

In conclusion, the here presented study has shown promising results regarding the adoption of phantomless calibration methodologies and provides reference density values for postmenopausal women to the scientific community which could be employed to carry out phantomless calibration when needed. These findings would not only offer potential benefits in enhancing the current clinical practice but would also allow the scientific community to take advantage of a higher number of CT scans, including opportunistic CT scans, to reliably build FE models. Such methodology could therefore support the adoption of digital twin technologies in clinics, fostering, *inter alia*, the creation of validation collections to increase the credibility evidence of CT-based digital twins.

## Supporting information

S1 File Detailed information about CT scans, phantom-based calibration, kernel distribution fitting and results. (PDF)
