# Supplementary material for: Phantomless calibration of CT scans for hip fracture risk prediction in silico: Comparison with phantom-based calibration
Source: PLoS One. 2024 Jun 14;19(6):e0305474. doi: 10.1371/journal.pone.0305474 (PMC11178222; doi:10.1371/journal.pone.0305474)
Supplement: S1 File — (PDF) [file pone.0305474.s001.pdf]

Phantomless calibration of CT scans for hip fracture risk prediction *in silico*: comparison with phantom-based calibration

Julia A. Szyszko, Alessandra Aldieri, Antonino A. La Mattina, Marco Viceconti

Detailed information about CT scans

Table S1. Detailed information of subjects CT scan. Subjects marked in green are the Group 1, and the white ones compose the Group 2.

Subject	Age	Height	Weight	Acquisition Year	CT settings of calibration	Spacing Between Slices (mm)	Slice Thickness (mm)	Tube Voltage (kVp)	Xray Tube Current (mA)	Kernel	Manufacturer	Manufacturer Model
S01	76	155	55	2006	4	2	3	120	190	Bone	GE MEDICAL SYSTEMS	HiSpeed CT/i
S02	75	160	65	2006	1	2	3	120	180	Bone	GE MEDICAL SYSTEMS	HiSpeed CT/i
S03	78	172	83	2006	3	2	3	120	170	Bone	GE MEDICAL SYSTEMS	HiSpeed CT/i
S04	60	167	63	2002	1	2	3	120	180	Bone	GE MEDICAL SYSTEMS	HiSpeed CT/i
S05	66	159	47	2003	1	2	3	120	180	Bone	GE MEDICAL SYSTEMS	HiSpeed CT/i
S06	58	180	88	2005	4	2	3	120	190	Bone	GE MEDICAL SYSTEMS	HiSpeed CT/i
S07	64	165	73	2007	3	2	3	120	170	Bone	GE MEDICAL SYSTEMS	HiSpeed CT/i
S08	56	160	78	2006	4	2	3	120	190	Bone	GE MEDICAL SYSTEMS	HiSpeed CT/i
S09	71	158	70	2006	1	2	3	120	180	Bone	GE MEDICAL SYSTEMS	HiSpeed CT/i
S10	57	152	76	2006	4	2	3	120	190	Bone	GE MEDICAL SYSTEMS	HiSpeed CT/i
S11	69	165	62	2023	5	1.25	1.25	120	100	Bone	GE MEDICAL SYSTEMS	Discovery CT
S12	82	160	50	2023	5	1.25	1.25	120	100	Bone	GE MEDICAL SYSTEMS	Discovery CT
S13	70	168	60	2023	6	1.25	1.25	120	140	Bone	GE MEDICAL SYSTEMS	Discovery CT
S14	66	169	54	2023	5	1.25	1.25	120	100	Bone	GE MEDICAL SYSTEMS	Discovery CT
S15	70	163	89	2023	6	1.25	1.25	120	140	Bone	GE MEDICAL SYSTEMS	Discovery CT
S16	79	160	67	2023	5	1.25	1.25	120	100	Bone	GE MEDICAL SYSTEMS	Discovery CT
S17	78	160	60	2023	5	1.25	1.25	120	100	Bone	GE MEDICAL SYSTEMS	Discovery CT
S18	77	165	39	2004	2	2	3	120	200	Bone	GE MEDICAL SYSTEMS	HiSpeed CT/i
S19	76	168	100	2006	2	2	3	120	200	Bone	GE MEDICAL SYSTEMS	HiSpeed CT/i
S20	71	160	59	2004	2	2	3	120	200	Bone	GE MEDICAL SYSTEMS	HiSpeed CT/i
S21	73	165	70	2005	2	2	3	120	200	Bone	GE MEDICAL SYSTEMS	HiSpeed CT/i
S22	84	150	64	2006	2	2	3	120	200	Bone	GE MEDICAL SYSTEMS	HiSpeed CT/i
S23	77	150	50	2005	2	2	3	120	200	Bone	GE MEDICAL SYSTEMS	HiSpeed CT/i
S24	67	160	56	2004	2	2	3	120	200	Bone	GE MEDICAL SYSTEMS	HiSpeed CT/i
S25	65	160	68	2001	2	1	3	120	200	Bone	GE MEDICAL SYSTEMS	HiSpeed CT/i

S26	56	158	62	2002	2	2	3	120	200	Bone	GE MEDICAL SYSTEMS	HiSpeed CT/i
S27	59	158	59	2000	2	1	3	120	200	Bone	GE MEDICAL SYSTEMS	HiSpeed CT/i
S28	76	150	67	2003	2	2	3	120	200	Bone	GE MEDICAL SYSTEMS	HiSpeed CT/i
S29	61	165	78	2003	2	2	3	120	200	Bone	GE MEDICAL SYSTEMS	HiSpeed CT/i
S30	67	154	73	2003	2	2	3	120	200	Bone	GE MEDICAL SYSTEMS	HiSpeed CT/i
S31	67	160	59	2003	2	2	3	120	200	Bone	GE MEDICAL SYSTEMS	HiSpeed CT/i
S32	64	152	45	2004	2	2	3	120	200	Bone	GE MEDICAL SYSTEMS	HiSpeed CT/i
S33	59	154	53	2005	2	2	3	120	200	Bone	GE MEDICAL SYSTEMS	HiSpeed CT/i
S34	75	160	70	2005	2	2	3	120	200	Bone	GE MEDICAL SYSTEMS	HiSpeed CT/i
S35	61	167	69	2005	2	2	3	120	200	Bone	GE MEDICAL SYSTEMS	HiSpeed CT/i
S36	61	143	68	2007	2	2	3	120	200	Bone	GE MEDICAL SYSTEMS	HiSpeed CT/i
S37	66	165	82	2006	2	2	3	120	200	Bone	GE MEDICAL SYSTEMS	HiSpeed CT/i
S38	57	157	80	2006	2	2	3	120	200	Bone	GE MEDICAL SYSTEMS	HiSpeed CT/i
S39	68	166	66	2005	2	2	3	120	200	Bone	GE MEDICAL SYSTEMS	HiSpeed CT/i
S40	68	160	69	2007	2	2	3	120	200	Bone	GE MEDICAL SYSTEMS	HiSpeed CT/i
S41	60	155	54	2000	2	1	3	120	200	Bone	GE MEDICAL SYSTEMS	HiSpeed CT/i

Detailed information about Phantom-based calibration

Table S2. Detailed information of ESP phantom scans with different configurations.

Calibration	Spacing between slices (mm)	Slice Thickness (mm)	Tube Voltage (kVp)	Xray Tube Current (mA)	Kernel	Manufacturer	Manufacturer Model	intercept	slope
1	4.5	3	120	180	Bone	GE MEDICAL SYSTEMS	HiSpeed CT/i	-0.004860	0.000812
2	4.5	3	120	200	Bone	GE MEDICAL SYSTEMS	HiSpeed CT/i	-0.004792	0.000806
3	4.5	3	120	170	Bone	GE MEDICAL SYSTEMS	HiSpeed CT/i	-0.002523	0.000806
4	4.5	3	120	190	Bone	GE MEDICAL SYSTEMS	HiSpeed CT/i	-0.000368	0.000799
5	1.25	1.25	120	100	Bone	GE MEDICAL SYSTEMS	Discovery CT	-0.011511	0.000816
6	1.25	1.25	120	140	Bone	GE MEDICAL SYSTEMS	Discovery CT	-0.010314	0.000810

Kernel distribution fitting procedure

Voxels in the ROI were used to build a histogram (bin width 1 HU), which was later fitted with a kernel distribution using a normal smoothing function with a 5 HU bandwidth (Fig S1). For that purpose, Matlab (release R2022b, The Mathworks Inc) built-in function *fitdist* was used.

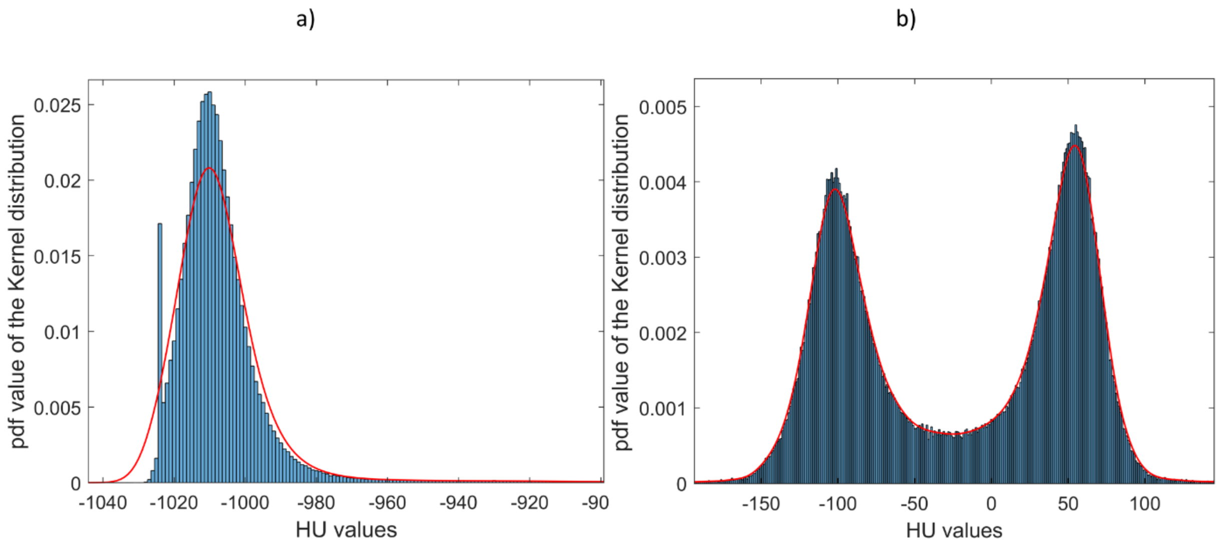

Fig S1. Example of histogram of ROI with fitted distribution for air (a) and adipose and muscle (b). The anomaly bin around HU = -1020 in subfigure (a) represents pixel values used in the image to pad to a rectangular format. These pixels are not part of the anatomical image.

The smoothing bandwidth was determined through convergence analysis of the obtained HU peak values, aiming to achieve an error smaller than 5 % (Fig S2).

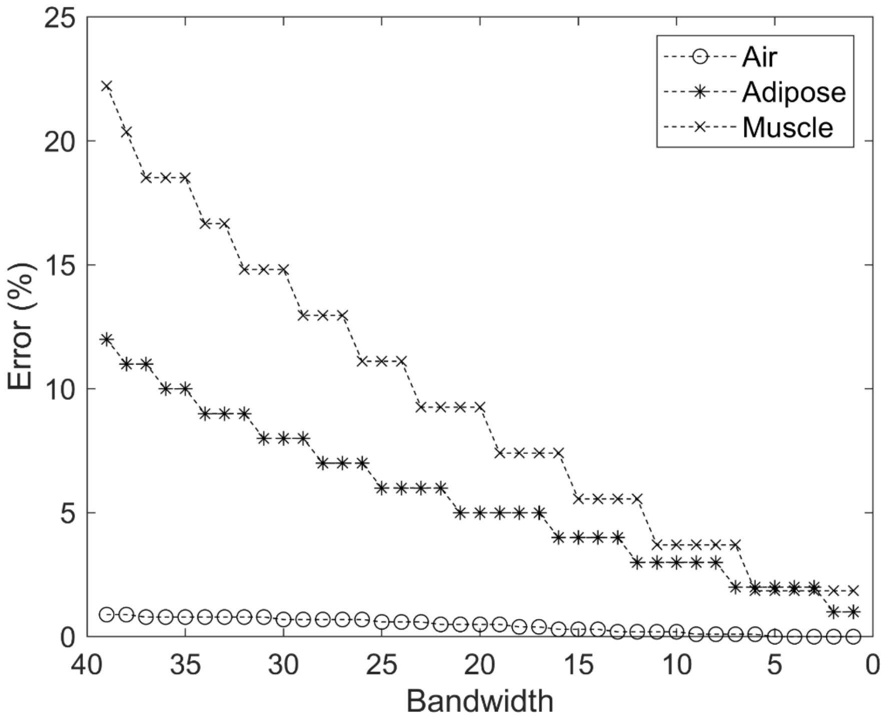

Fig S2. Convergence analysis for different bandwidth values. Error was computed on obtained HU values for air, adipose and muscle tissue for each bandwidth to the values obtained for the lowest one.

Young’s modulus analysis

Fig S3 presents violin plots of the element-by-element relative differences in the Young’s modulus resulting from the two calibration methodologies for all the subjects in the Group 2. Notably, the high relative differences were associated with low density values (Fig S4).

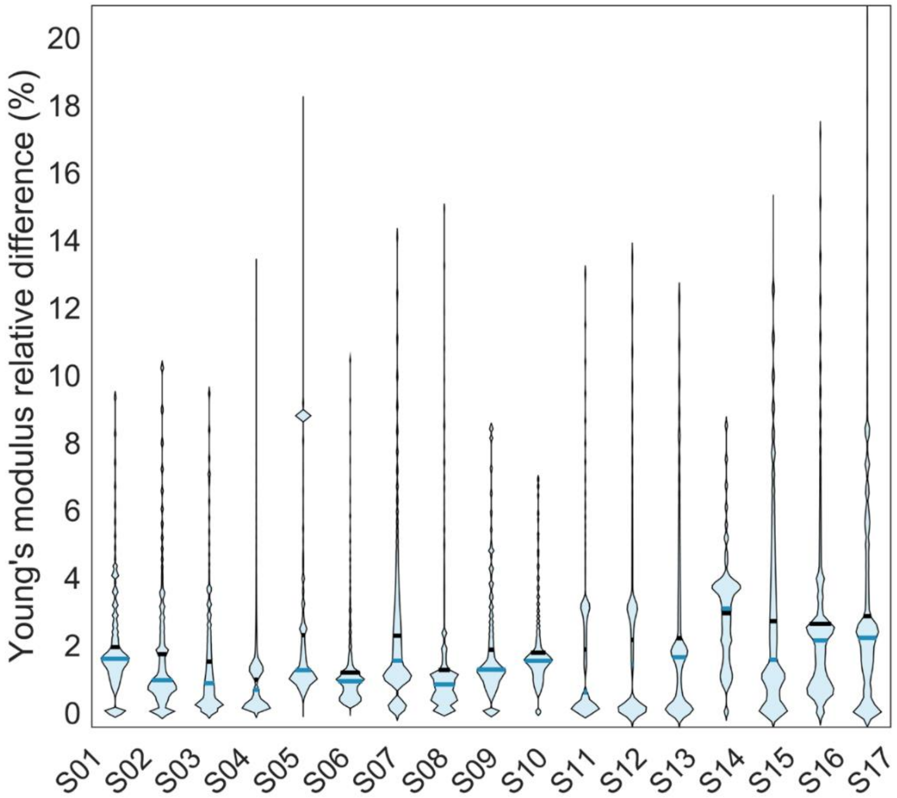

Fig S3. Distribution of element-wise relative differences in Young’s modulus between both calibration methods. Violin plots showing the distributions of element-wise relative differences in Young’s modulus values between phantom-based and phantomless calibration for Group 2 subjects. The solid black line represents the mean value, while the blue solid line represents the median.

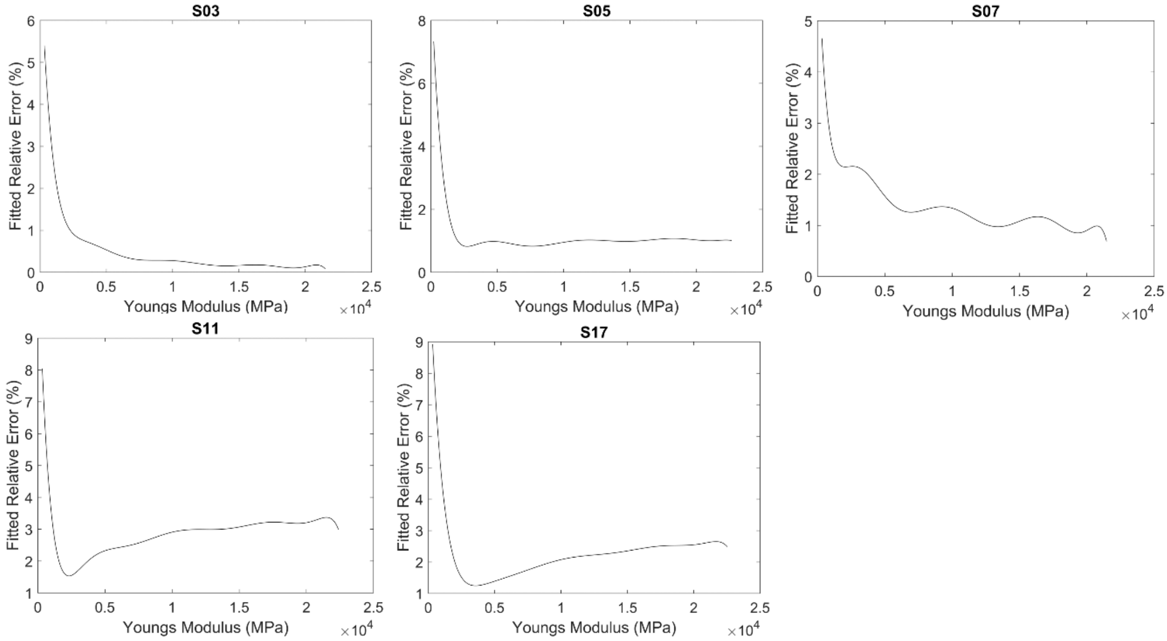

Fig S4. The distribution of the relative errors (%) across Young's modulus values for selected subjects.

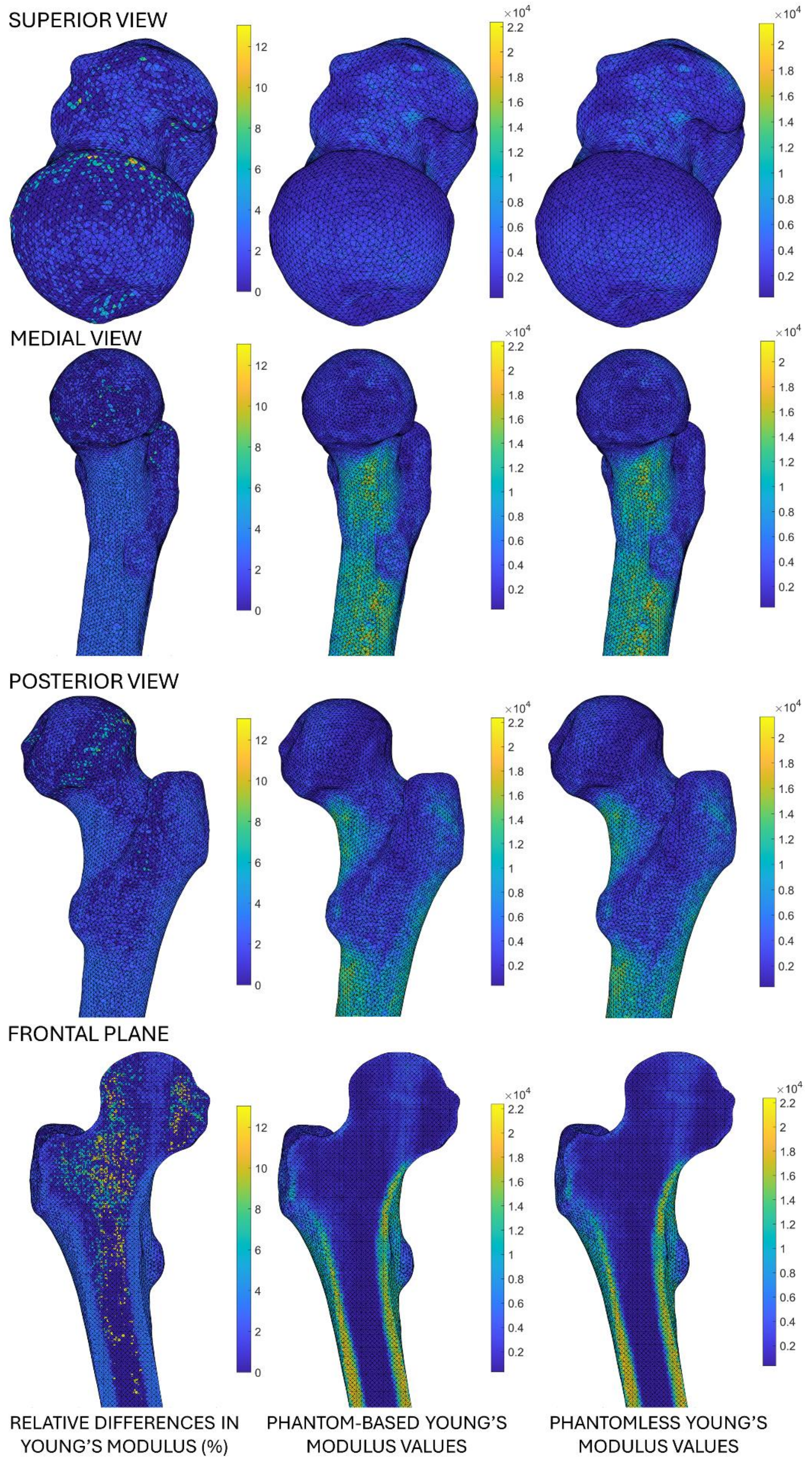

Fig S5. Spatial distribution of relative differences between Young's modulus values coming from the phantom-based and phantomless calibrations for S11. Frontal plane is the mean frontal section from the anterior view of the femur.

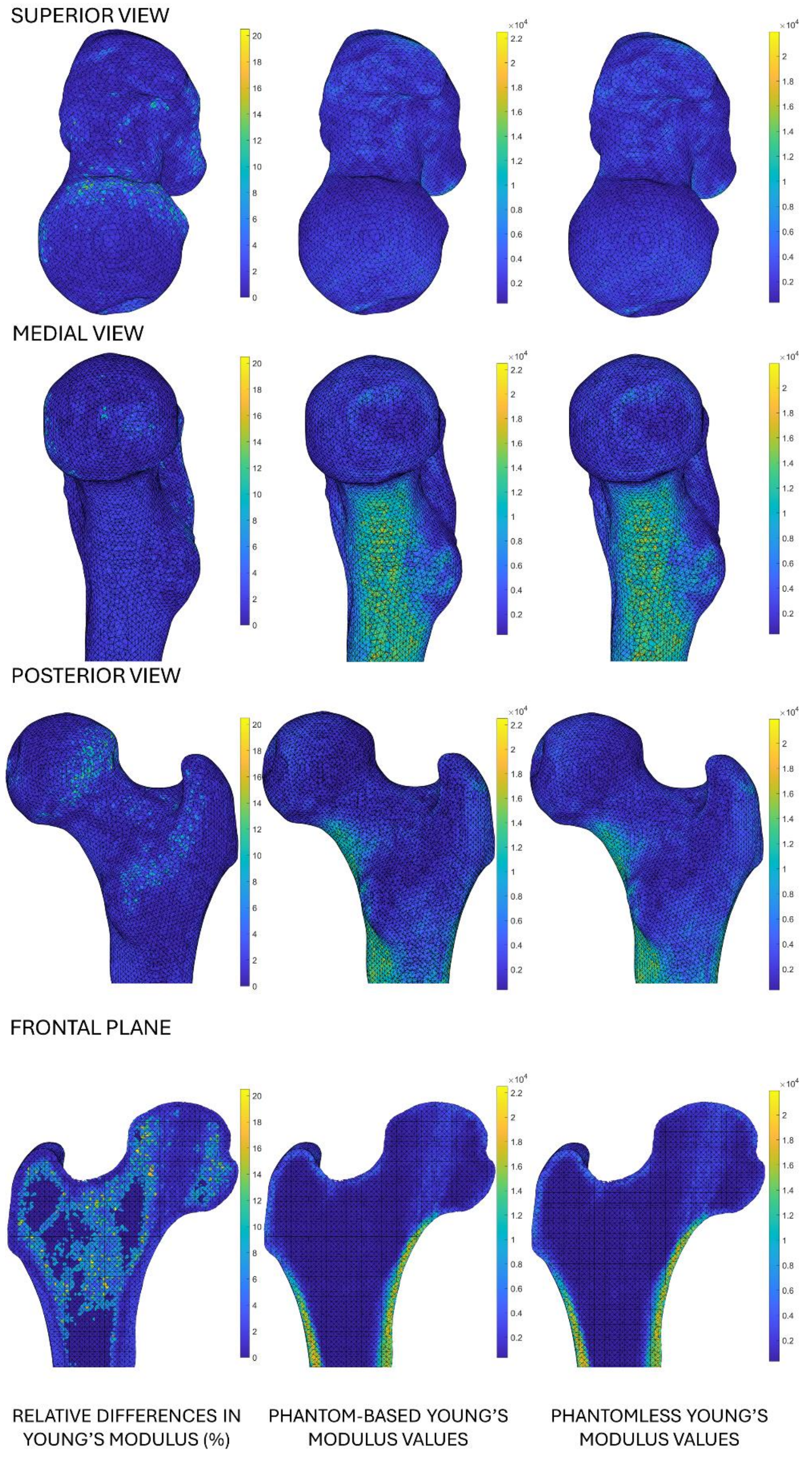

Fig S6. Spatial distribution of relative differences between Young's modulus values coming from the phantom-based and phantomless calibrations for S17. Frontal plane is the mean frontal section from the anterior view of the femur.

Tensile principal strain analysis

Average point-to-point relative differences for tensile strains and root mean square relative error are shown in Fig S7 and Fig S8.

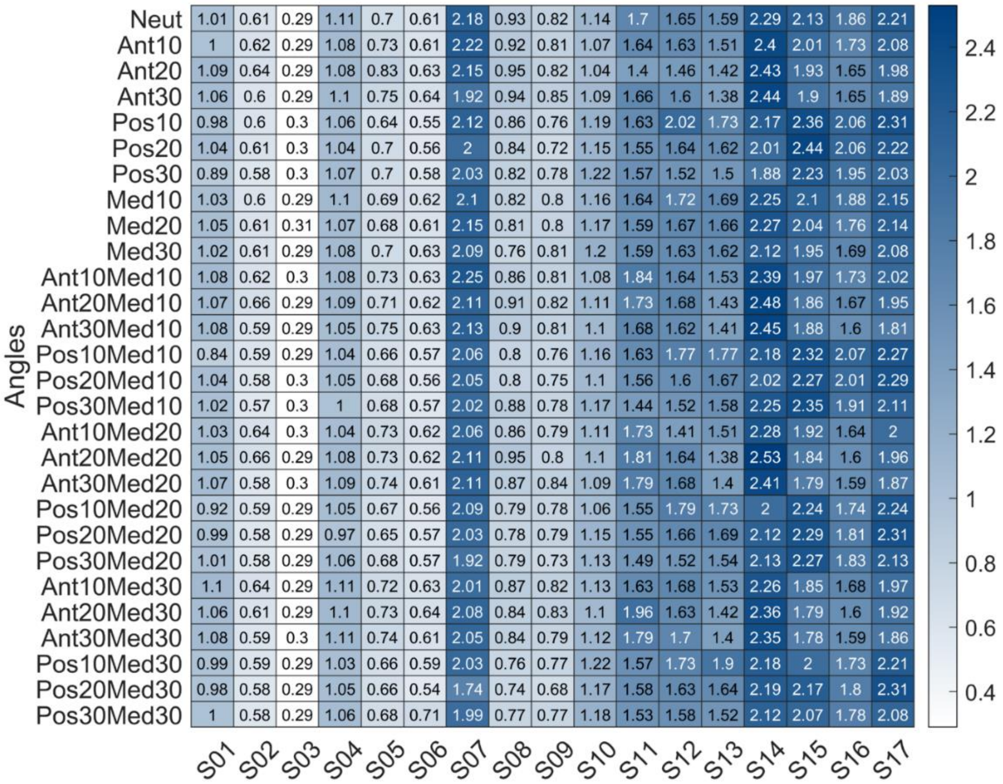

Fig S7. Average point-to-point relative differences (%) in superficial tensile strains between the phantom-based and phantomless methods.

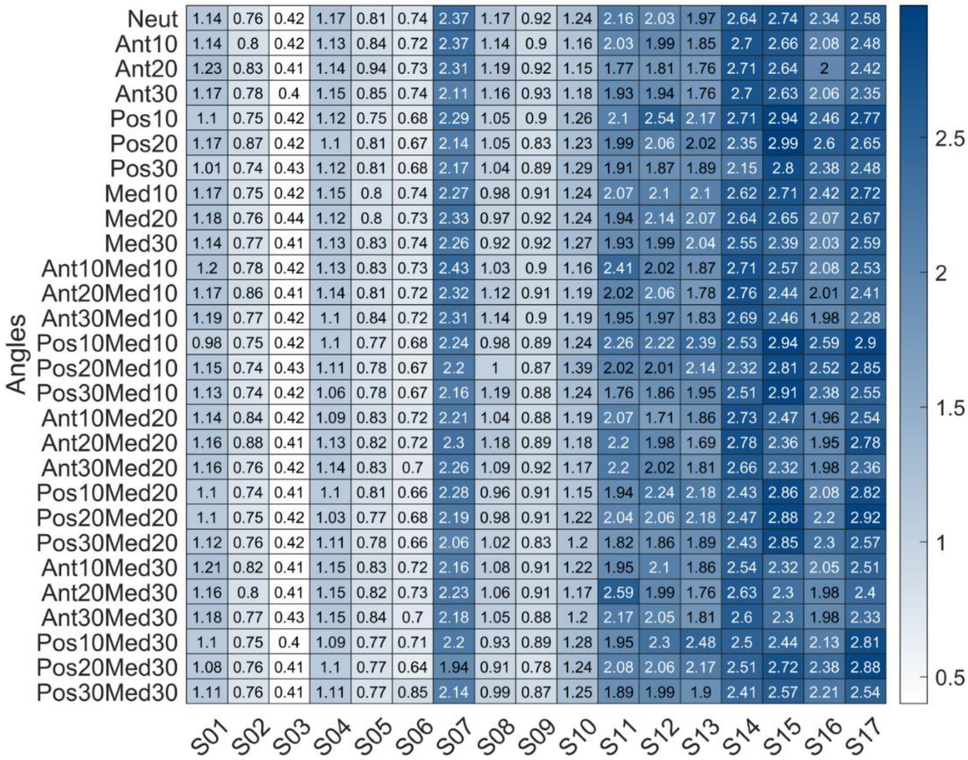

Fig S8. Root mean square relative error (%) in superficial tensile strains between the phantom-based and phantomless methods.

Compressive principal strain analysis

Average point-to-point relative difference for compressive strains and root mean square relative error are shown in Fig S9 and Fig S10.

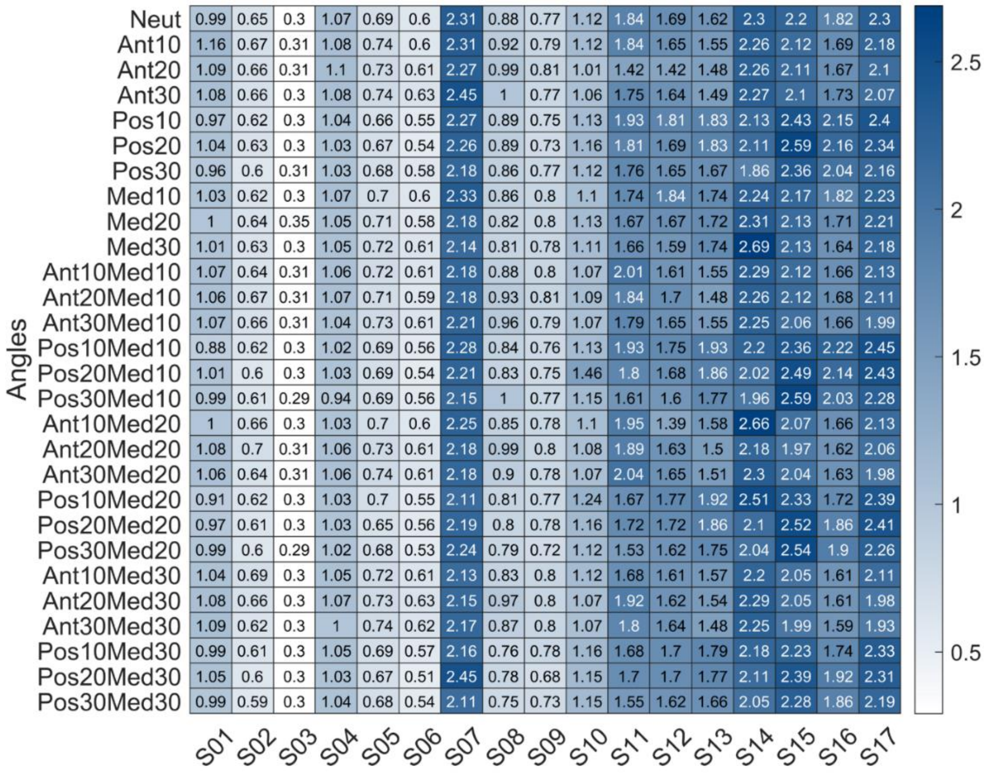

Fig S9. Average point-to-point relative differences (%) in superficial compressive strains between the phantom-based and phantomless methods.

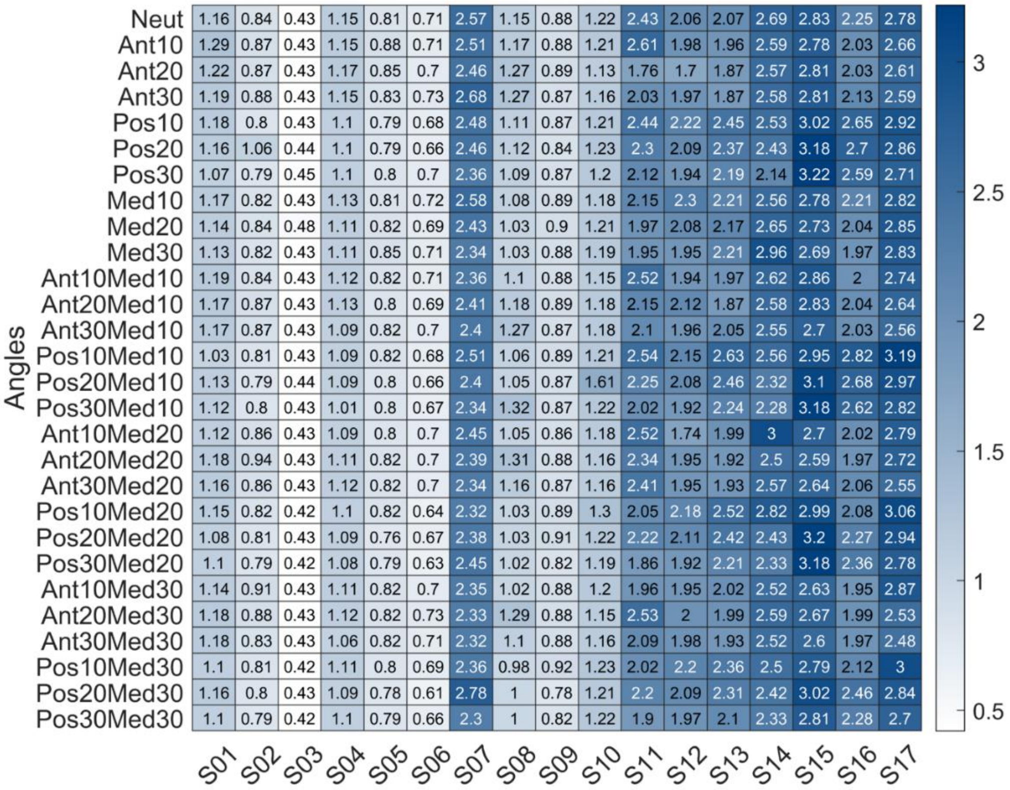

Fig S10. Root mean square relative error (%) in superficial compressive strains between the phantom-based and phantomless methods.

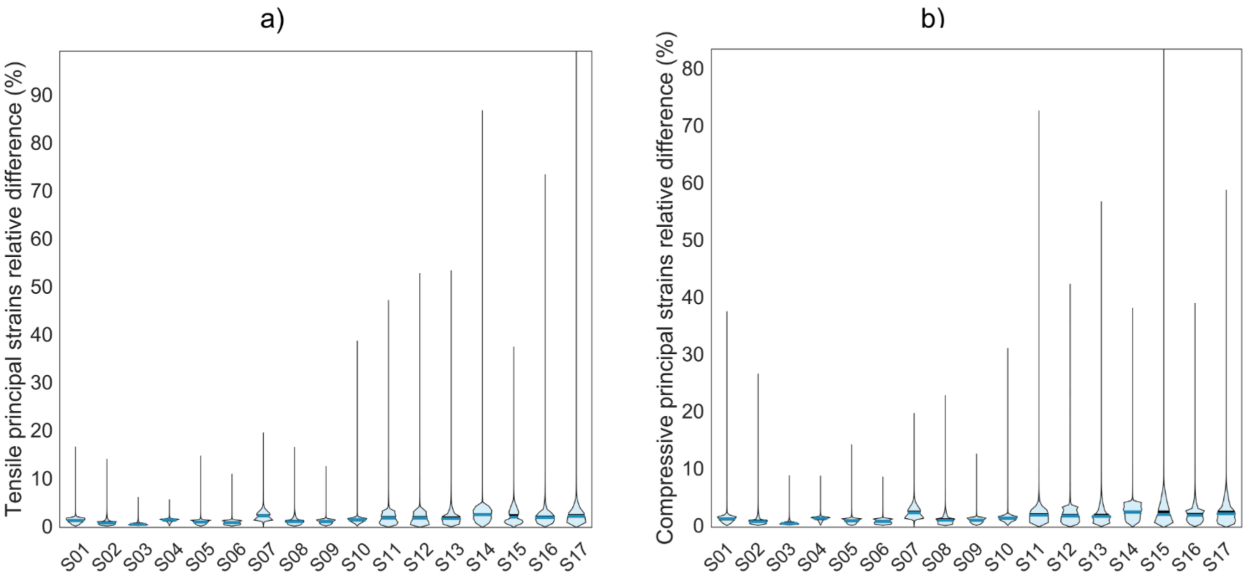

Fig S11. Distribution of point-to-point relative differences in superficial principal strains coming from both calibration methods. Violin plots showing the distributions of the point-to-point relative differences computed on superficial tensile (a) and compressive (b) principal strain values between phantom-based and phantomless calibrations, considering all 28 simulated femur impact poses for each the 17 subjects in Group 2. The solid black line represents the mean, the blue solid line represents the median.

Minimum Side-Fall Strength analysis

Relative error on Minimum Side-Fall Strength (MSF), *i.e.*, the lowest load to failure across the 28 femoral impact poses between phantom-based and phantomless calibration method for each subject from Group 2 is shown in Table S3.

Table S3. Comparison of phantom-based and phantomless failure loads, angles, and relative errors for each subject in Group 2.

Subject	Phantom-based				Phantomless				
	MSF	Failure Load Angle	Intra-Extra rotation angle	Abduction angle	MSF	Failure Load Angle	Intra-Extra rotation angle	Abduction angle	Relative Error on Failure Load (%)
S01	1143.07	Pos20Med30	Extra20	30	1149.97	Pos20Med30	Extra20	30	0.6
S02	1478.35	Pos30Med30	Extra30	30	1479.61	Pos30Med30	Extra30	30	0.08
S03	1694.55	Ant30Med30	Intra30	30	1696.84	Ant30Med30	Intra30	30	0.14
S04	1975.12	Pos20Med30	Extra20	30	1956.89	Pos20Med30	Extra20	30	0.93
S05	1966.79	Pos30Med30	Extra30	30	1957.43	Pos30Med30	Extra30	30	0.48
S06	2195.41	Pos10Med30	Extra10	30	2202.4	Pos10Med30	Extra10	30	0.31
S07	1583.99	Ant20Med30	Intra20	30	1546.19	Ant20Med30	Intra20	30	2.41
S08	1634.78	Ant20Med30	Intra20	30	1612.79	Ant20Med30	Intra20	30	1.35
S09	1745.72	Pos30Med30	Extra30	30	1734.65	Pos30Med30	Extra30	30	0.64
S10	2113.47	Ant20Med30	Intra20	30	2131.34	Ant20Med30	Intra20	30	0.84
S11	2054.84	Pos30Med30	Extra30	30	2036.43	Pos30Med30	Extra30	30	0.90
S12	1166.30	Ant10Med30	Intra10	30	1171.46	Ant10Med30	Intra10	10	0.44
S13	1181.04	Ant30Med10	Intra30	10	1194.77	Ant30Med10	Intra30	30	1.16

S14	2357.00	Ant30Med30	Intra30	30	2321.63	Ant30Med30	Intra30	30	1.50
S15	1740.50	Pos30Med30	Extra30	30	1782.02	Pos30Med30	Extra30	30	2.39
S16	2181.24	Pos30Med30	Extra30	30	2202.54	Pos30Med20	Extra30	20	0.98
S17	1594.42	Ant20Med30	Intra20	30	1617.78	Ant20Med30	Intra20	10	1.46

ARF0 analysis

The obtained values of ARF0 for each subject in Group 2 for both calibration methods, along with the absolute differences are shown in Table S4.

Table S4. Comparison of phantom-based and phantomless ARF0 values, along with absolute differences.

Subject	ARF0 Phantom-based (%)	ARF0 Phantomless (%)	Absolute Difference (%)
S01	26.35	25.74	0.60
S02	33.99	33.71	0.28
S03	30.10	29.89	0.21
S04	22.67	23.60	0.94
S05	13.96	14.43	0.47
S06	15.62	15.43	0.19
S07	23.37	25.24	1.87
S08	7.69	8.15	0.47
S09	1.15	1.21	0.07
S10	0.06	0.05	0.01
S11	26.18	26.62	0.44
S12	32.63	32.75	0.12
S13	37.27	36.08	1.19
S14	14.66	16.42	1.76
S15	0.93	0.71	0.22
S16	8.84	8.68	0.16
S17	28.87	27.90	0.97

Prior analysis

While employing the method with the reference density values proposed in Eggermont *et al.* (2019), we obtained notable errors in Young's modulus, tensile (ε_1) and compressive (ε_3) principal strains shown in the Table S5. The table reports errors computed with respect to the phantom-based calibration in terms of both the root mean square relative error (RMSRE) and relative differences in Young's modulus, tensile (ε_1) and compressive (ε_3) principal strains for the 10 subjects belonging to Group 2 (S01-S10).

Table S5. Errors obtained while employing Eggermont *et al.* (2019) method.

	RMSRE (%)			Relative Differences (%)		
	mean	std	max	min	max	mean
ε_1	3.21	4.51	16.42	5.15	9.52	7.38
ε_3	10.57	1.81	16.78	4.93	9.43	7.31
Young's modulus	12.69	1.96	16.21	5.89	8.01	6.92

References:

Eggermont, F., et al. (2019). Calibration with or without phantom for fracture risk prediction in cancer patients with femoral bone metastases using CT-based finite element models. PLOS ONE, 14(7):e0220564.
